# Enhancing Prenatal Group Medical Visits with Mindfulness Skills: A Pragmatic Trial with Latina and BIPOC Pregnant Women Experiencing Multiple Forms of Structural Inequity

**DOI:** 10.1007/s12671-023-02227-z

**Published:** 2023-10-10

**Authors:** Larissa G. Duncan, Na Zhang, Trilce Santana, Joseph G. Cook, Lisabeth Castro-Smyth, Margaret S. Hutchison, Tuyen Huynh, Deena Mallareddy, Laurie Jurkiewicz, Nancy Bardacke

**Affiliations:** 1 School of Human Ecology, University of Wisconsin—Madison, Madison, WI, USA; 2 Human Development & Family Sciences, University of Connecticut, Storrs, CT, USA; 3 Osher Center for Integrative Health, University of California San Francisco, San Francisco, CA, USA; 4 Obstetrics, Midwifery, and Gynecology Clinic, Zuckerberg San Francisco General Hospital and Trauma Center, San Francisco, CA, USA; 5 Department of Psychology, University of South Carolina, Columbia, SC, USA; 6 Mindful Birthing and Parenting Foundation, Oakland, CA, USA

**Keywords:** Pregnancy, Mindfulness, Group prenatal healthcare, Postpartum depression, Spanish

## Abstract

**Objectives:**

Prenatal mindfulness programs can improve mental health, yet access to and cultural and linguistic relevance of existing programs in the United States are limited for people who do not speak English and/or face major life stressors such as migration, housing instability, limited income, and racism. In response, mindfulness skills training drawn from Mindfulness-Based Childbirth and Parenting (MBCP) was integrated into Medicaid-covered CenteringPregnancy (CP) group prenatal healthcare, delivered in Spanish and English by certified nurse-midwives and community co-leaders, and tested in a pragmatic pilot trial.

**Method:**

A provider survey of 17 CP clinics informed development of the enhanced program. Next, it was tested with 49 pregnant people who chose CP prenatal care. All of the sample identified as women; 4% as LGBTQ +; 90% as Black, Indigenous, and People of Color (65% as Latina/e/x); 10% as White; and 63% as Spanish-speaking. Groups were allocated 1:1 to CenteringPregnancy or CenteringPregnancy with Mindfulness Skills (CP +).

**Results:**

Intent-to-treat analysis of self-report interview data indicated CP + yielded lower postpartum depression (the a priori primary study outcome) with a large effect size (Cohen’s *d* = 0.80) and a trend toward lower postpartum anxiety (Cohen’s *d* = 0.59) compared to CP. Hypothesized effects on mindfulness, positive/negative affect, and perceived stress were only partially supported at post-birth follow-up. Satisfaction with care was high across conditions.

**Conclusions:**

Augmenting group prenatal healthcare with mindfulness training in Spanish and English appears feasible, did not reduce satisfaction with care, and may have additional mental health benefits. Key questions remain about structural supports for perinatal well-being.

A growing body of research indicates participation in prenatal mindfulness training is related to myriad benefits for perinatal mental health and well-being (e.g., Corbally & Wilkinson, 2021; Leng et al., 2023; Shi & MacBeth, 2017; Yan et al., 2022), yet it is not widely accessible nor viewed as culturally relevant for many communities most in need of support in the United States (U.S.). Pregnant people with high socioeconomic status (SES) and racial and linguistic privilege (e.g., White English-speakers) can typically access supplemental stress reduction and mental health care, such as mindfulness training, on their own (Olano et al., 2015). Many people from global majority communities, however, may experience structural, cultural, and linguistic barriers to integrative healthcare access in predominantly White institutions in the U.S. Vulnerable and underserved populations, often overrepresented by Black, Indigenous, and People of Color (BIPOC) community members due to structural racism, may also lack resources (e.g., time, income, insurance coverage) needed to access supplemental care, which contributes to persistent racial/ethnic health disparities.

Mindfulness-based programs (MBPs) tailored specifically for expectant families have been adapted from Mindfulness-Based Stress-Reduction (MBSR: Kabat-Zinn, 1990) and Mindfulness-Based Cognitive Therapy (MBCT; Segal et al., 2004) programs, with accumulating evidence showing a variety of beneficial impacts for participants (e.g., Dimidjian et al., 2015). One such model that uses a childbirth education approach is the Mindfulness-Based Childbirth and Parenting program (MBCP; Bardacke, 2012). MBCP has been shown to improve maternal mental health during pregnancy and postpartum in comparison to other high-quality hospital and community-based childbirth education courses in randomized controlled trials (RCTs) in the U.S. (Duncan et al., 2017; Sbrilli et al., 2020), Sweden (Lönnberg, et al., 2020, 2021), and Hong Kong (Zhang et al., 2023), among others. One study of an adapted MBCP program demonstrated long-term effects on depression compared to treatment as usual that were maintained 8 years following the pregnancy intervention (Roubinov et al., 2022). A recent RCT in China demonstrated a reduction in stress and improvement in hypothalamic-pituitary-axis function for MBCP participants compared to active controls (Wang et al., 2023). Although MBCP is currently available in numerous countries, its delivery outside of research contexts in the U.S. has been largely limited to English-speaking populations who typically have higher income and educational opportunity. A notable exception is a Federally Qualified Health Center (FQHC) in the Northeastern region of the U.S. serving an immigrant and refugee population where MBCP groups have been implemented with success (Moffit, 2017).

An initial pilot study of MBCP (Duncan & Bardacke, 2010) documented that pregnant women in the San Francisco (SF) Bay Area who choose this program for their childbirth education are predominantly White, U.S.-born English speakers, and well-resourced in terms of SES and healthcare access, with most receiving prenatal care through private insurance or private pay and who intentionally sought out MBCP to address their stress and fears related to pregnancy and childbirth. In contrast, a study of immigration status and use of health services among Latina women in the SF Bay Area (Fuentes-Afflick and Hessol, 2009) showed that nearly three quarters of these women had immigrated to the U.S., over half were without documentation, 62% did not have a primary healthcare provider, 40% were uninsured, and approximately 33% had no preventive health visits in the previous year. In a focus group study of mental health services as part of prenatal care for Black women in the SF Bay Area (Kemet et al., 2022), participants identified significant, and often insurmountable, barriers to receiving mental health care during pregnancy. One participant “… managed her anxiety and depression before pregnancy without therapy or medication but found that the added stress of pregnancy worsened her symptoms past the point she could manage alone” (Kemet et al., 2022; pp. 781). Yet despite being covered by government-sponsored insurance, receiving a referral from her prenatal provider, and considerable effort on her part, she was unable to access mental health services during pregnancy. Women in Kemet’s focus groups were receptive to the idea of group prenatal care as a vehicle for mental health support.

As suggested by these examples, and copious epidemiological data, advances in prevention and health promotion strategies are urgently needed at multiple levels to promote perinatal health equity in the U.S. (Gennaro et al., 2020). Group medical visits (GMVs) are one promising avenue for improving perinatal outcomes in an accessible model. A major advantage of GMV models for prenatal healthcare is their potential to make medical care that is reimbursable through private insurance or Medicaid more inclusive and culturally relevant in ways that benefit patients and may further impact clinicians and healthcare systems (Carter et al., 2021). GMVs allow providers to cover vastly more health education in a roughly equivalent amount of patient contact hours; for patients, GMVs transform 1.5–2 hr of prenatal care into 15 to 20 hr of care. For example, instead of attempting to discuss the same prenatal nutrition guidelines or postpartum contraception options with each patient in a series of brief 15- to 20-min individual visits, a group of pregnant people meet together with their provider for a 2-hr session with ample time for discussion regarding numerous relevant and timely topics. In addition, GMVs may lead to cost savings compared to individual care as found in a study of Medicaid supported *CenteringPregnancy* in South Carolina (Gareau, et al., 2016).

Beyond increasing health knowledge during pregnancy at a rate 3 times greater than individual care (Ratzon et al., 2022), the *CenteringPregnancy* (CP) model of group prenatal care is designed to provide healthcare utilization empowerment and social support. An RCT of CP showed a 33% overall reduction in risk of preterm birth (PTB), with an even larger reduction of PTB risk for Black women, and greater rates of breastfeeding initiation overall (Ickovics et al., 2007). Recent trials of CP show mixed results, with no overall PTB reduction, but better birth outcomes linked with higher participation in group care, particularly for Black participants (Crockett et al., 2022). Black people in the U.S. are the racial group experiencing the worst racial disparities in preterm birth, low infant birthweight, and maternal morbidity and mortality (Osterman et al., 2023; Carty et al., 2022), disparities that worsened during the COVID-19 pandemic (U.S. Government Accountability Office, 2022) and persist across socioeconomic lines (Magesh et al., 2021). CP or other community-based GMVs that are even more culturally relevant may be an essential approach for this population. For Latina women, research on CP has shown greater likelihood of receiving adequate care (Tandon et al., 2013), higher satisfaction with care (Robertson et al., 2009), and better birth outcomes (Tandon et al., 2012; Trudnak et al., 2013).

The mechanisms whereby group prenatal care achieves its effects have not been thoroughly investigated (Massey et al., 2006; Rising et al., 2004) and there may be room for improvement in these domains through the addition of mindfulness practices to reduce stress and promote prenatal and postpartum mental health and well-being. To fill this gap, mindfulness skills training drawn from MBCP was integrated into CP group prenatal healthcare covered by Medicaid (government provided insurance) and private health insurance. Pre-pilot work included (a) a provider survey of 17 CenteringPregnancy sites to determine need for and receptivity to this approach; and (b) a curriculum development pilot whereby the MBCP program developer participated in CP sessions to facilitate MBCP content and discussion topics. A curriculum workgroup included the MBCP and CP developers, the study researchers, and CP providers where a consensus was reached on the approach. The resulting MBCP enhanced *CenteringPregnancy with Mindfulness Skills* (CP +) was then delivered by a safety net hospital credentialed providers in a community setting in Spanish and English, and tested in a small pragmatic, quasi-experimental pilot trial.

The current study addresses the first aim of the trial by testing whether CP + had additive effects on self-reported psychological functioning in the prenatal and postpartum period compared to a time and attention-matched CP control group. It was hypothesized that the CP + group would (a) experience less postpartum depression (the a priori primary outcome), and (b) as secondary outcomes demonstrate greater increases in mindfulness, positive emotion, and coping; greater reductions in pregnancy anxiety, negative emotion, perceived stress, and depression symptoms from the first to the third trimester of pregnancy; and healthier psychological functioning postpartum compared to the standard CP group. The secondary outcomes were hypothesized to be potential mechanisms of action that could link CP + participation with better birth outcomes in future large-scale trials. Using pragmatic trial principles (Patsopoulos, 2022), we also investigated feasibility and acceptability of the additions to CP in a real-world setting, recruiting participants to join the research study after they were already enrolled in care. Since CP is a well-established and effective model, we tested for equivalence across conditions in satisfaction with care.

## Method

### Participants

Potential participants aged 18 to 35 were recruited from among those pregnant people enrolled to receive group-based prenatal healthcare from the safety net public hospital midwifery clinic’s CenteringPregnancy program delivered in a community setting near the hospital. At the start of the study, 90% of the hospital’s patient population was designated as low-income and participant healthcare was covered by the California state-specific Medicaid program, Medi-Cal. Some CP sites use an “opt-in” model where pregnant people must choose CP in lieu of individually delivered prenatal care. Other CP sites use an “opt-out” model where pregnant people are enrolled in CP care unless they choose not to proceed with group medical visits for their prenatal care. Our study partner site used an opt-out model. People with high-risk pregnancies (i.e., pregnancies with a co-occurring medical condition with increased risks to the health or life of the pregnant person, the fetus, or both) (NICHD, n.d.) are offered individually delivered care (e.g., through high-risk obstetrics clinics) to offer closer monitoring. Our partner site permitted high-risk participants to enroll in CP while also receiving 1:1 visits with specialized providers to augment their group care as long as their condition did not interfere with CP group participation.

**Fig. 1:**
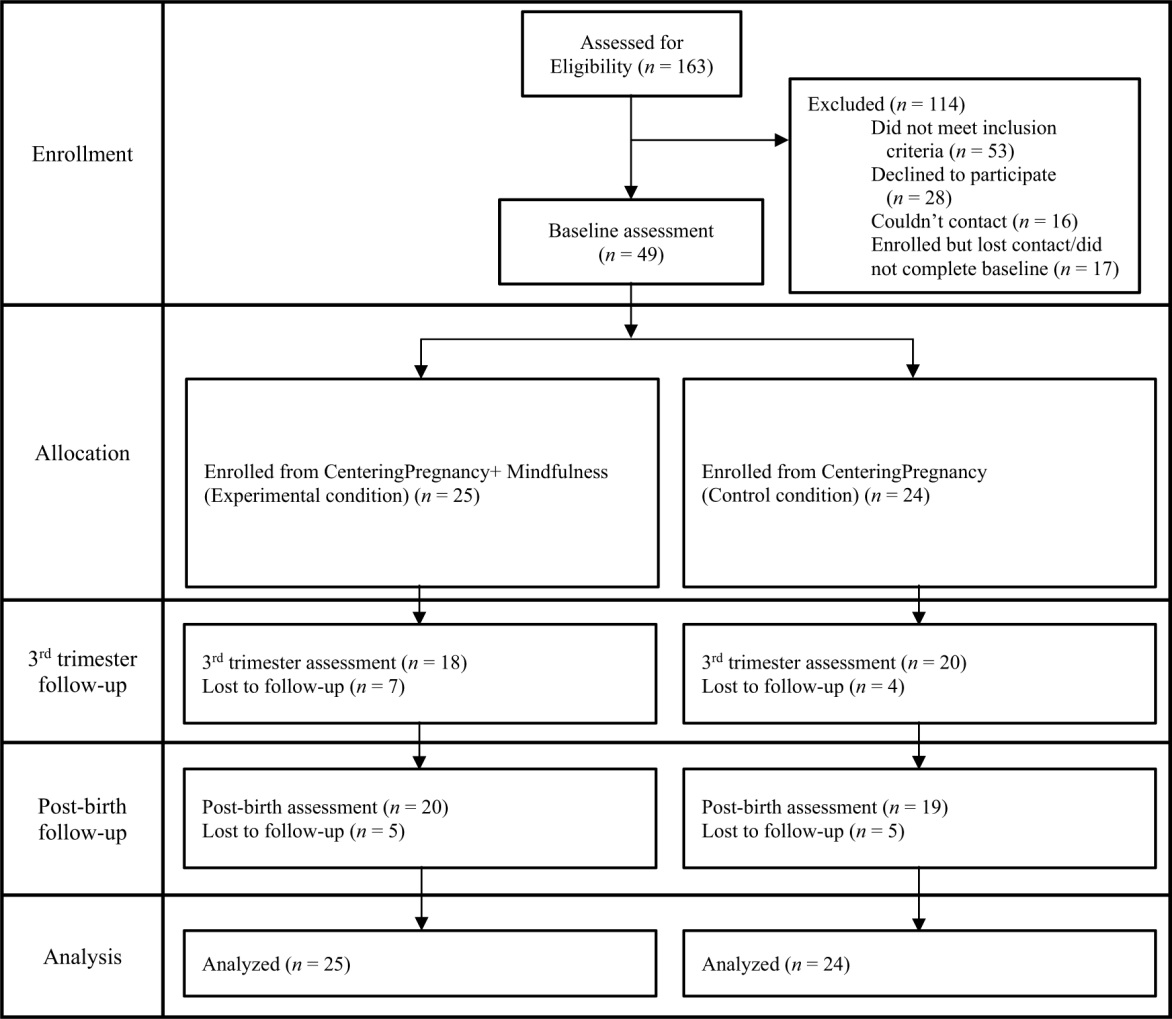
CONSORT participant flow chart

Both primiparous and multiparous pregnant people were included in the study who were enrolled in CP, able to communicate verbally in Spanish and/or English, and planned a hospital birth in the geographic area. They were excluded from the study if they had type 2 diabetes, HIV, seizure disorder, serious mental health disorder, substance abuse, or another medical condition that would lead to an inability to follow Centering program guidelines; were not able to communicate in English or Spanish; had previously participated in CP; or had formal training in meditation, yoga, or other mind–body practices. A total of 163 patients who were enrolled in CP by our partner site during the study recruitment period were referred to the study for screening and *n* = 49 met inclusion/exclusion criteria and were successfully enrolled in the study out of a target sample size of 72 (target *n* = 60 after attrition). See Fig. 1 for the CONSORT participant flow diagram.

After group allocation, there were *n* = 24 participants in the CenteringPregnancy group (CP: the active, time- and attention-matched control condition) and *n* = 25 participants in the CenteringPregnancy with Mindfulness Skills group (CP + : the experimental condition). There were *n* = 31 participants (63.2%) who selected Spanish as the language for their research interviews, and *n* = 18 participants (36.7%) who selected English. For race/ethnicity, 65.3% (*n* = 32) identified as Latino(a)/Latin American/Hispanic; 10.2% (*n* = 5) as White/European American, not of Hispanic origin; 8.2% each as Indigenous/Native American/Alaskan Native/American Indian (*n* = 4); Black/African American (*n* = 4); and multi-racial/mixed race (*n* = 4). The full sample (*n* = 49; 100%) identified as women and a majority (*n* = 47; 95.9%) identified as heterosexual or straight; one person identified as gay or lesbian; and one person identified as bisexual. Sixty-five percent of participants were born in a country other than the U.S. with *n* = 15 (30.6%) from México, one from Europe, two from South America, and the rest from countries of Central America (Table 1). Number of years living in the U.S. ranged from 1 to 22 (*M* = 6.63, *SD* = 4.98).

Thirty-one participants (63.3%) reported living in their own apartment or house, whereas the remaining 18 (36.7%) were experiencing homelessness (*n* = 2) or another unstable living situation (*n* = 16), such as a hotel where they paid by the month or renting a room in an apartment with other people. Twenty-one (42.8%) participants reported between 4 and 11 years of formal education. Sixteen (32.6%) reported annual family income of less than US$10,000/year and 14 (28.6%) as between US$10,001 and $30,000, far below the city’s median household income of US$76,963 (Federal Reserve Bank, n.d.) during the study. A majority had Medicaid coverage (*n* = 45; 92%); and 14 (28.6%) were receiving EBT/food stamp support. See Table 1 for more details.

### Procedures

All study procedures were approved by the university IRB, the hospital CRC review board, and the NIH/NCCIH Office of Clinical and Regulatory Affairs. We also obtained a Certificate of Confidentiality from the National Institutes of Health to provide an added layer of protection for participants without documented immigration status.

#### Study Design

The original proposal included a randomized controlled trial (RCT) design with random assignment of individuals to groups and an explanatory research focus. Our partner site, the midwifery service of the safety net public hospital informed us that it was not possible to randomize individuals given the frequency, volume, and structure of the ongoing CenteringPregnancy groups offered by their service. Pregnant people in CP are grouped based on their month of due date and there were not enough people receiving care with the same month due date to randomly assign individuals to groups. Instead, we adopted a more pragmatic, quasi-experimental approach whereby pairs of groups were assigned to a condition in a 1:1 distribution. For example, for two Spanish-language CP groups beginning in July and August, one group received CP + and one group received CP. English-language groups were just beginning at the site and had a wider spread of due dates than typically recommended in CP. The decision regarding which group would be held as CP + was predetermined based on these schedules; the condition for a given month’s group was concealed from nurses who initially enrolled the participants in care. The groups of participants were not expected to differ in any meaningful way beyond what was expected by chance.

Participants were informed that there were differences between the two conditions regarding the stress reduction components; however, they were not told the specific differences between the conditions. Both intervention groups were presented in an even-handed manner, and efforts were made to reduce participants’ perception of one group as more desirable. Several additional steps were taken to minimize the potential effects of knowledge of group assignment in influencing outcomes. The intention was for group assignment to be concealed from the research staff who conducted participant interviews. In practice, the follow-up interviews included questions about CP + mindfulness elements, revealing group assignment.

#### Recruitment

A medical history and physical examination were conducted by one of the certified nurse-midwives (CNMs) at intake into prenatal healthcare. Staff at the midwifery service informed eligible people about the research study during their intake visit. Clinic staff used chart review to pass names, addresses, email addresses, phone numbers, and eligibility criteria (age/birth date and due date/gestational age) of participants in eligible CP groups on to the study staff. Study staff who were fluent in Spanish and English contacted potentially eligible participants by phone, email, and/or mail to recruit them into the study.

#### Baseline Visit

After a phone screen of CP enrolled prenatal healthcare clients referred by the midwifery clinic, research staff invited those who were eligible for the study to schedule a baseline appointment. If possible, the consent form was mailed to the potential participant for review prior to the scheduled appointment. This visit took place at the hospitalbased Clinical Research Center (CRC). Informed consent and questionnaire assessment (and psychophysiological assessments, a component of the broader trial not addressed here) were conducted at the baseline visit in the language of choice of the participant (Spanish or English).

#### Informed Consent

Written informed consent was obtained from all study participants before performing any study procedures. The study staff offered to read the IRB-approved consent and HIPAA forms to the participant and answered any questions.

#### Incentives

Transportation vouchers for public transportation to the hospital CRC were provided to participants for each visit, along with snacks during the study visit. Incentives for time spent in assessment (approved by the IRB) were provided in the form of cash or money order for each study visit, totaling US$150 per participant if all assessments were completed. Participants were not compensated for CP/CP + attendance.

#### Questionnaire Administration

The questionnaires were administered by trained interviewers in the language of choice of the participant (Spanish or English) using Computer-Assisted Personal Interview (CAPI; QDS Data Systems) and visual aids with response options for Likert-type scales and the social ladder. CAPI allows collection of high-quality interview data in a standardized fashion and data entry needs.

#### Follow-up Visits

Participants were asked to complete two follow-up visits: one during the 3rd trimester of pregnancy (28 to 32 weeks gestational age) and one postpartum (6 to 10 weeks post-birth). The protocol for the 3rd trimester visit was identical to the baseline visit (other than the elimination of some demographic items in the interview that would not change) and took place at the CRC. For the postpartum assessment, participants were given the option of (a) coming to the CRC, or (b) having the research assistant visit them in their home or another suitable location to administer the assessment. This was intended to promote retention due to the difficulty new parents may face in being away from home in the early postpartum period. The research staff had the appropriate child abuse clearances and background check with fingerprinting to allow them to assist with infant care as needed during the postpartum visit.

#### Assessment Schedule

All 49 participants completed a baseline assessment at time A, *n* = 38 participants completed the assessment at time B, and *n* = 39 participants completed the assessment at time C. Participants were evaluated at (1) baseline prior to attending their 2nd centering session (gestational age in weeks: *Range* = 13.71 to 27.57 weeks; *M* = 19.67, *SD* = 3.85); (2) in the 3rd trimester (gestational age in weeks: *Range* = 28.43 to 38.86 weeks; *M* = 31.80, *SD* = 2.49); and (3) 6 to 20 weeks after the final centering session (weeks post birth: *Range* = 5.71 to 23.14 weeks; *M* = 12.67, *SD* = 4.50).

#### Interventions

*CenteringPregnancy* (CP) prenatal healthcare visits (medical appointments) take place in groups, where 8–12 pregnant people (who have due dates in approximately the same calendar month) meet together with their credentialed healthcare provider (CNM or obstetrician) and another trained group facilitator (e.g., medical assistant, social worker) for a 2-h period. Thus, they are essentially pooling their medical visit minutes with a group of other pregnant people with a similar due date and engaging in group discussion regarding health education topics, pregnancy, and childbirth-related issues, versus doing so individually. Visits follow the practice guidelines from the American College of Obstetrics and Gynecology for prenatal healthcare. All sessions of CP include three core components of (1) health assessment (including blood pressure, weight, and fundal height assessment), (2) interactive learning, and (3) community building.

CP content topics for group discussion include nutrition, exercise, relaxation, understanding pregnancy problems, infant care and feeding, postpartum issues including contraception, comfort measures in pregnancy, sexuality and childbearing, abuse issues, parenting, and childbirth preparation. CP is a healthcare empowerment model where pregnant people learn to take and chart their own blood pressure and weight measurements, discuss their own health concerns as well as knowledge, and support one another during the perinatal period, all with the guidance of providers in a nonhierarchical approach. CP is focused on honoring group process and dynamically responding to the topics that arise as most salient for the participants; thus, specific content topics in the curriculum may be addressed in different sessions as long as they are covered at some point in the 10 sessions, requiring the provider to be organized but flexible (Rising, n.d.). The CP model provides local sites and providers with flexibility to incorporate cultural adaptations and modify implementation elements while maintaining adherence to the essential elements of CP. Some CP sites include partners or support people, others do not, limiting participation to the pregnant person only. Our partner site warmly welcomes partners/support people’s attendance and active participation in CP group sessions, while being sensitive to potential challenges with their inclusion (e.g., if there is abuse occurring in the relationship). Due to poverty- and immigration-related life challenges, only a small proportion of partners/support people attend, typically between 0 and 5 for a group of 8 to 12 pregnant participants.

##### CenteringPregnancy with Mindfulness Skills

(CP +) consists of the CenteringPregnancy approach and content combined with mindfulness skills training incorporated in each session adapted from the Mindfulness-Based Childbirth and Parenting (MBCP) program. MBCP for expectant parents is a 9-week, 3-h per session childbirth education course that includes a daylong silent meditation retreat that is an adapted version of the Mindfulness-Based Stress Reduction (MBSR) Program. See Bardacke (2012) and Duncan & Bardacke (2010) for a detailed description of the MBCP curriculum. MBCP is offered to pregnant people and their partners/support people and includes mindfulness meditation on the breath, body, feelings, thoughts, and emotions; a body scan meditation; yoga postures practiced with mindful awareness of the body and the physical changes associated with pregnancy; a “Being with Baby Practice” to promote prenatal attachment; an adapted loving-kindness meditation; and preparation for mindful parenting in the postpartum period. In addition, MBCP includes specific mindfulness practices for coping with stress, pain, and fear associated with pregnancy, childbirth, and early parenting with a focus on shifting the way participants relate to physical pain, negative thoughts, and emotions and cope with stress in everyday life. See Table 2 for a list of MBCP components that were included in CP + in an English-language group. CP has an implicit emphasis on mindful listening and responding through “facilitative leadership” which was made more explicit in CP +. CP + providers were also mentored around intentionally highlighting mindfulness threads during group conversations. Given the dynamic nature of CP group sharing, Table 2 provides just one example of the order in which the adapted MBCP content could be woven into CP +. MBCP and CP + practices designed for dyads can be done with pairs of pregnant participants in lieu of partner participation.

#### Curriculum Development Process

To develop the combined curriculum of CP enhanced with MBCP skills training, two pre-pilot studies were conducted. First, a survey was conducted with 17 CenteringPregnancy sites in the broader geographic region, both in hospital settings and in community clinics, to obtain CP provider perspectives on (a) the main sources of stress faced by pregnant people in their CP groups; (b) how well providers are able to address patient stress within the standard CP model (5-point Likert-type scale ranging from *Poorly* to *Very Well*); (c) whether they felt there was room for improvement in the CP model regarding stress reduction (*Yes*, *No*, *Unsure*); and (d) potential areas for improvement (open ended). Additional survey items included questions about patient demographics, the function and sustainability of CP at their site, and provider characteristics, to inform the goal of making the adapted curriculum relevant for the patient and provider populations using CP.

Each site was asked to identify two key CP providers to complete the survey. Thirteen sites responded to the survey, with a total of *n* = 24 individual respondents, including 9 CNMs and 4 family physicians. Most respondents reported serving a predominantly low-income patient population with 95–100% of patients identified as low-income. Providers reported a variety of sources of patient stress. The most common of these were financial pressures (instability, limited resources), immigration-related concerns (legal status, isolation from family in country of origin), relationship issues (tension, domestic violence), and anxiety related to the current pregnancy and other medical concerns. When asked how well the CP model addresses stress, 33% of respondents said, *Very Well*; 25% said, *Well*; 12.5% said, *Somewhat*; and one said, *Not Well*. Not all survey respondents answered this question. Many providers reported discussing stress management during CenteringPregnancy groups, yet 50% of the respondents felt there was room for improvement. Meditation, yoga, and creative arts (music, crafts) were listed as potential stress reduction activities for inclusion in the CP model.

Overall, providers reported that CP participants experience stress from an array of sources in multiple domains of life. Half of CP providers felt that the CP model allows them to adequately address stress reduction among their patient populations and the other half did not. Mind–body stress reduction modalities were listed as potential areas for enhancing the CP model.

Following indications from the survey that an enhancement to CP would be potentially desirable and beneficial, the MBCP developer (a CNM and experienced meditation teacher) partnered with an experienced CNM CP provider and attended two full rounds of CP delivered in English. In the first round, in order to become familiar with the CP model, the MBCP developer was a participant-observer. In the second round of CP, in collaboration with the study team, the MBCP developer and CP provider piloted the inclusion of adapted MBCP content into CP in an iterative manner. They observed participant engagement, noted participant questions, and attended to reports of benefits from and barriers to the practices. Subsequently, a series of curriculum workgroup meetings were convened with the study team, intervention developers, and CNM providers. The PI, having completed the initial level of CP facilitator training and bringing a background of professional training in MBSR and MBCP, oversaw the curriculum development process. Consensus was reached among the curriculum workgroup regarding the approach for this trial regarding the CP + enhancement.

#### Community-Based Setting

For the current study, the CP/CP + groups were held at a community-based organization (CBO) near the public safety net hospital. The CBO provides wraparound services related to food security, housing, immigration support, and other instrumental needs, often with culturally and linguistically congruent providers. Partnering with the CBO added an accessibility dimension intended to increase healthcare and public service utilization and provided a space conducive to CP group delivery consistent with the CP model (e.g., a room with pleasant lighting and décor large enough for the group to sit in a circle).

#### Spanish Language and Cultural Adaptation

The third author and fifth author have professional expertise as Spanish-language translators and interpreters and were responsible for Spanish-language translations of (a) study assessment tools (e.g., qualitative interview protocols) and (b) MBCP curriculum additions to CP +, including scripts for the guided audio recordings of mindfulness practices. They paid special attention to the countries of origin most common among the Spanish-speaking immigrant population in the geographic region (Mexico and countries of Central America) and worked to ensure culturally relevant word choices. Because some participants were Indigenous immigrants (e.g., from Maya communities) who primarily spoke Indigenous languages and Spanish secondarily, efforts were made to increase ease of comprehension through a “plain language” (National Institutes of Health, n.d.) approach. They also worked with the PI and the MBCP developer on the CP + curriculum development to help identify and culturally adapt mindfulness practices that may have disturbing connotations for participants that could be experienced as racist microaggressions. For example, we renamed a mindful movement exercise with the arms reaching overhead “reaching for the stars” that was previously described as “picking grapes” that invoked imagery too closely aligned with lived experiences of some participants involved in the highly stressful and often inhumane conditions of migrant farm work. The foundation of the curriculum was the CP model that had already undergone a deep cultural adaptation by the partner site to be appropriate for delivery to Latina/e/x participants. The CP model offers flexibility for local adaptation to promote cultural relevance.

#### Provider Qualifications

The midwifery service had CenteringPregnancy site approval from the Centering Healthcare Institute (CHI; www.centeringhealthcare.org). CNMs who provided CP in the study had completed CP facilitator training through CHI and had extensive experience delivering the CP model. In some cases, they also served as national CHI faculty members providing training and conducting site approval visits with other sites in the U.S. Group co-leaders were most often employees of the CBO where groups were held, and some were graduates of CP and/or other CBO programs and services. Co-leaders were also trained and experienced in the CP model.

CNMs who provided CP + completed professional training in MBCP with the MBCP program developer at the university home for the study. CP + providers were supported in developing or continuing a personal mindfulness practice and encouraged to take MBSR if they had not already done so. The MBCP developer and the PI, both White women with decades of personal meditation experience and basic Spanish language skills, provided ongoing mentoring to the CP + providers during the study. CNM providers identified as White women and spoke Spanish with varying degrees of fluency. Interpreters were available in the CP/CP + groups, a role sometimes held by the CBO-based group co-leader.

### Measures

In most cases, self-report measures used in the study were previously validated in English and Spanish. One measure, Cumulative Experiences of Multiple Forms of Oppression, investigated only as a potential covariate here was newly created (by the fifth and first authors) and translated into Spanish for this study (by the fifth author).

#### MacArthur Scales of Subjective Social Status

The MacArthur SSS Scale was developed by Adler and team (1994; 2000), as a single item used to assess how a person perceives their relative rank in comparison to others in their group. Participants are shown a drawing of a ladder with 10 rungs. They then read or hear that the ladder “represents where people stand in society” and that “At the top of the ladder are the people who are the best off, those who have the most money, most education, and best jobs. At the bottom are the people who are the worst off, those who have the least money, least education, worst jobs, or no job. Please place an ‘X’ on the rung that best represents where you think you stand on the ladder.” In the current study, two versions of the SSS scale ladder were used, each producing a score ranging from 0 to 10: (a) a ladder for ranking their status in relation to their own community, and (b) in relation to everyone in the U.S. This measure has been validated in Spanish, and a study with Latinx immigrants showed that it predicted a range of health outcomes better than a standard income measure (Garza et al., 2017). Another study with Mexican immigrants living on a low income demonstrated prediction of health outcomes even after controlling for objective SES (Franzini & Fernandez-Esquer, 2006),

#### Cumulative Experiences of Multiple Forms of Oppression

This measure is comprised of three sets of items. The first set is comprised of 20 items that assess experiences of discrimination in the past year with response options ranging from 0 = *Never* to 4 = *Almost Every Day*. Participants are asked to rate how often each has happened to them because of unfair treatment on the basis of their race, ethnicity, gender, nationality, language, religion, income level, education level, sexual orientation, age, disability, illness, height, weight, or other personal characteristics. Example items include “Being treated with less respect than other people,” “Being treated suspiciously, monitored, or followed while in public places,” “Being unfairly stopped, questioned, physically threatened or abused by police or other authorities,” “Having your partner rejected or ignored, having your relationship denied or not acknowledged,” and “Being denied health care or provided inferior medical care.” The second set of 7 items assesses how often participants have felt a sense of isolation, fear for their safety, and other negative emotions due to unfair treatment in the past year with response options ranging from 0 = *Never* to 4 = *Very Often*. These two subscales are scored by taking a mean of the items. Cronbach’s alphas in the current study were 0.86 for the *experiences* subscale and 0.85 for the *feelings* subscale. The third set of items assesses lifetime cumulative experiences of unfair treatment based on each possible identity category (listed above), including an open-ended item for other identities, producing a frequency. Cumulative Experiences of Multiple Forms of Oppression (CEMFO) items were adapted from an array of other measures designed to assess racism and race-related stress (Ibañez et al., 2009; Jackson et al., 2005; Ramirez-Valles et al., 2010; Utsey & Ponterotto, 1996; Yoo et al., 2010).

#### Depression

To assess the primary outcome of the current study, the 10-item Edinburgh Postnatal Depression Scale (EPDS; Cox et al., 1987) was administered at postpartum follow-up. The EPDS was summed and used as a continuous variable and a dichotomous variable with a cut-point as an indication of potential clinical levels of postpartum depression. The EPDS has been validated in Spanish in Mexico (Flom et al., 2018) and Spain (Vázquez & Míguez, 2019). In clinical settings, cut-points of 10, 11, and 12 are used with varying degrees of sensitivity and specificity (with sensitivity declining at higher cut-points and specificity declining at lower cut-points) (Levis et al., 2020).

The 20-item Center for Epidemiologic Studies Depression Scale (CES-D; Radloff, 1977) was used to measure depressive mood at all three visits. Items are rated on a 4-point scale according to frequency of experience over the previous week. Items are summed yielding a score ranging from 0 to 60. Higher scores indicate higher levels of depression. We examined the CES-D both as a continuous variable to see the full range of depression symptoms including subclinical levels, and as a dichotomous variable with scores of 16 or greater indicating potential clinical levels of depression. Cronbach’s alpha in the current study was 0.87.

*Pregnancy anxiety* was assessed with the revised Pregnancy Anxiety Scale (Levin, 1991) containing 10 items regarding the degree of anxiety the mother feels during pregnancy about her own health: “I am worried about developing medical problems during my pregnancy”; the health of her developing fetus: “I have a lot fear regarding the health of my baby”; and healthcare during parturition: “I am afraid that I will be harmed during delivery.” Participants respond how often they have these thoughts and feelings (1 = *Never*; 5 = *Always*). The Pregnancy Anxiety scale had good internal consistency (⍺ = 0.80).

Postpartum anxiety was assessed with the widely used State-Trait Anxiety Inventory (STAI; Spielberger et al., 1970). State and trait dimensions are rated on a 4-point scale (*Almost Never* to *Almost Always*).

#### Mindfulness

The Five Factor Mindfulness Questionnaire (FFMQ) (Baer et al., 2006) was used to assess mindfulness. Participants are asked to indicate agreement (1 = *Never or Very Rarely True* to 5 = *Very Often* or *Always True*) with a list of 39 statements about their general tendency to be mindful in experiences of daily life. Examples items are as follows: “I pay attention to how my emotions affect my thoughts and behavior,” “I think some of my emotions are bad or inappropriate and I shouldn’t feel them.” The FFMQ has been shown to have adequate to good internal consistency (subscale alphas = 0.75 to 0.91) and convergent and discriminant validity in meditating and non-meditating samples (Baer et al., 2006). It was scored as an overall mean mindfulness score that had an overall Cronbach’s alpha of 0.80 in the current study.

#### Positive and Negative Emotion

The Differential Emotions Scale (DES; Izard, 1993), modified, was used to assess the frequency of positive and negative affect during the previous week. This version of the DES was modified by Fredrickson (Fredrickson et al., 2003) to include additional positive affect items yielding 28 total items. The scale is scored for total positive and negative affect. The mDES had acceptable reliability for the positive emotions subscale (⍺ = 0.83) and the negative emotions subscale (⍺ = 0.80) in the current study.

*Perceived stress* was measured with the 10-item version of the Perceived Stress Scale (PSS; Cohen & Williamson, 1988). This scale was designed for use with community samples and is now the most widely used self-report measure of psychological stress. Participants respond how often (1 = *Never*; 5 = *Very Often*) during the past month they experienced thoughts and feelings such as “felt that you were unable to control the important things in your life,” “been unable to control irritations in your life.” Cronbach’s alpha in the current study was 0.87.

*Coping* was assessed with a subset of items from the “Ways of Coping” (WOC; Folkman et al., 1986). The WOC is among the most widely used coping inventories. Participants were asked to respond how often they use specific ways of coping in relation to their self-reported stressful event of pregnancy (1st and 3rd trimesters) and parenting (postpartum). Additional items were added to gauge whether participants used specific mind–body coping skills taught in the interventions to cope with stress, for a total of 32 items.

### Data Analyses

#### Preliminary Analyses

We tested baseline group differences on all outcome variables of interest, and there were no statistically significant differences (*p*-values > 0.05). We tested baseline group differences on participants’ age, gestational age at baseline in weeks, interview language of choice, country of origin, family income, education, subjective socioeconomic status, lifetime cumulative experiences of multiple forms of oppression, and housing status (1 = *experiencing homelessness or another type of unstable living arrangement*; 0 = *living in an apartment or house*). There were no significant differences between the intervention and control groups except for family income.

Participants in the CP + experimental condition had higher income than participants in the control condition (*M*_CP+_ = 1.44 [*SD* = 1.20] vs. *M*_CP_ = 0.64 [*SD* = 0.85], *p* < 0.05). See Table 1 for the scale and range. Family income was added as a covariate to all analyses for hypothesis testing.

Bivariate correlations showed a few statistically significant associations between covariates and outcome variables: Subjective social status in relation to their community was positively associated with dispositional mindfulness at post-birth (*r* = 0.35); lower education was associated with greater likelihood of meeting diagnostic criteria for EPDS postpartum depression (*r* = −0.33) and CES-D depression symptoms at post-birth (*r* = −0.35); lower income (*r* = −0.46) and experiencing homelessness or other unstable living arrangements (*r* = 0.43) were also associated with a greater likelihood of meeting diagnostic criteria for CES-D depression symptoms at post-birth. These covariates were tested in additional models to the analyses for the specific outcome variables reported below.

To test the hypotheses that CenteringPregnancy with Mindfulness Skills would show greater benefits than the CenteringPregnancy program on indicators of mental health and psychological well-being, regression models were used to compute the group difference (CP + = 1; CP = 0). Specifically, for the hypothesized primary outcome of postpartum depression (EPDS), group assignment and family income were included in the models (education was also added for the binary outcome of EPDS). Because the EPDS is a postpartum measure, baseline depression symptoms assessed with the CES-D were used as a covariate for postpartum depression assessed with the EPDS.

For the secondary outcomes, dispositional mindfulness (FFMQ), depression symptoms (CES-D), anxiety (the trait anxiety subscale of the STAI), perceived stress (PSS), negative and positive emotions (mDES), mind–body coping strategies (WOC), and pregnancy anxiety (PAS), group assignment, family income, and baseline levels of the outcome variable were included, except for post-birth anxiety (STAI), for which baseline pregnancy anxiety (PAS) was used as a covariate (because STAI was not measured at baseline). Subjective social status in relation to their community was also added for the model of dispositional mindfulness FFMQ. Education and housing status were also added for the model of binary outcome of CES-D.

If an overall group difference was not detected, moderation effects were tested by calculating an interaction term (baseline level by group assignment) to examine whether group differences were dependent on baseline levels of the outcome. Screening of normality of data for dependent variables found that skewness and kurtosis were all acceptable (< 2 for skewness, < 7 for kurtosis; West et al., 1995). All assumptions for regression were met.

#### Missing Data

Key study variables had between 18 and 24% missingness in the data. Little’s MCAR test (Little, 1988) on all study variables did not reject the assumption of missingness at random (*χ*^2^ = 34.53, *DF* = 28, *p* = 0.18). Thus, an intent-to-treat approach was applied such that all participants’ data was analyzed, and missing data was handled with Full Information Maximum Likelihood using Mplus 8.4 (Muthén & Muthén, 2017).

## Results

The hypothesis that CenteringPregnancy with Mindfulness Skills (CP +) would show greater benefits than CenteringPregnancy in reducing postpartum depression was supported. While controlling for baseline depression symptoms (CES-D) and family income, there was a statistically significant effect of group assignment on postpartum depression levels assessed with the EPDS (unstandardized coefficient *B* = −3.04, *p* < 0.05, standardized coefficient β = −0.31). Parents in CP + had lower EPDS postpartum depression (*M* = 4.90; *SD* = 4.06) than their peers in the CP program (*M* = 8.68; *SD* = 5.39), Cohen’s *d* = 0.80 (e.g., a large effect).

To provide an estimation of clinical significance in addition to statistical significance, we also examined clinical cut-points for the EPDS (≥ 10 and ≥ 11). Using the cut-point of 10 and above on the EPDS to maximize sensitivity and avoid false negatives (Levis et al., 2020), 10% of the CP + group (*n* = 2 of 20) and 42.1% of the CP group (*n* = 8 of 19) met the criteria for possible clinical depression. The chi-square test of group differences on the dichotomous indicator was significant (*p* < 0.05); however, when controlling for income and education as well as baseline CES-D score, there was a marginally significant effect of group condition on EPDS diagnosis outcome (cutoff ≥ 10), *B* = −6.185, *p* = 0.051, odds ratio = 0.002, 95% *CI* = [0.000, 1.018].

In addition, while controlling for baseline pregnancy anxiety and family income, there was a marginally significant effect of group assignment on anxiety measured at post-birth (*B* = −4.49, *p* = 0.085, β = −0.26). There was a trend showing that participants in CP + had lower anxiety post-birth (*M* = 35.80; *SD* = 7.81) than their peers in CP (*M* = 40.79; *SD* = 9.06), Cohen’s *d* = 0.59 (i.e., a medium effect) (see Table 3 for means and standard deviations of study variables).

The secondary hypotheses regarding group differences in dispositional mindfulness, depression symptoms, perceived stress, positive and negative emotions, and mind–body coping strategies, all measured in the 3rd trimester and post-birth, were not supported at either the 3rd trimester or post-birth time points (*p*-values > 0.05; e.g., when interaction terms were not included). Thus, moderation effects were further tested at post-birth that found several statistically significant group differences moderated by baseline levels (see Supplemental Materials with plots of the moderation effects). To summarize, parents in the CP + group showed greater improvements in dispositional mindfulness, depression symptoms, perceived stress, and negative emotions at post-birth than mothers in CP if they had higher baseline levels of mindfulness, or lower levels of depression symptoms, perceived stress, or negative emotions, respectively. This suggests that the CP + enhancements were more beneficial for people with better psychological well-being regarding these outcomes compared to CP.

As an indicator of feasibility and acceptability, we examined attrition across groups and found a difference of only 1 more participant lost to follow-up in the CP group compared to the CP + group, suggesting the CP + enhancements did not differentially influence retention. In the 3rd trimester and post-birth assessments, CP + participants were asked to rate on a 4-point Likert-type scale from *Strongly Disagree* to *Strongly Agree* whether each MBCP practice helped them feel less stress. On average, participants rated every practice as helpful across both timepoints with the highest rating for the in-session mindfulness practices in the 3rd trimester (*M* = 2.47, *SD* = 0.72) and the lowest (though still positive) rating for the body scan at post-birth (*M* = 2.07; *SD* = 0.59) (Table 4). Qualitative reports regarding what they found most helpful about the sessions matched the CP + enhancements, e.g., staying calmer and more present in body and mind during pregnancy, staying calm when their baby is crying, noticing baby’s subtle cues, and practicing self-compassion in parenting (a key facet of mindful parenting; Duncan et al., 2009) by recognizing “That there’s no perfect way to do it” (Table 4).

## Discussion

The current study addressed the primary aim of a small trial intended to pilot a mindfulness enhancement to an existing model of group prenatal care, CenteringPregnancy, that was developed with input from CP providers. It was conducted in a community-based partnership with a safety net public hospital and a community-based organization serving pregnant people experiencing homelessness and housing instability. In this context, CenteringPregnancy was compared with a CP + version augmented with content adapted from Mindfulness-Based Childbirth and Parenting with involvement of the developers of both evidence-based intervention models. In a test of group differences on postpartum depression (PPD), participation in the CP + group was shown to be more beneficial, with lower rates of PPD than the CP group, with a large effect size. Thus, support was found for the primary self-report outcome of the trial specified in advance. One of the most robust outcomes of MBPs, including MBCP and other prenatal MBPs, is a reduction in depression (Leng et al., 2023; Yan et al., 2022). CP is also a robust model with available evidence showing many benefits, including some indication of potential impact on depression among pregnant adolescents (Felder et al., 2017). Contrasting the two versions in a time and attention-matched comparison was intended to isolate the additive benefit of the enhancement of GMVs with MBCP skills training. The lower rate of postpartum depression we found for CP + at post-birth follow-up combined with a trend toward lower postpartum anxiety partially supported study hypotheses regarding impacts on mental health. Trend level effects were expected with our limited sample size and require future investigation in larger trials.

Rates of PPD across conditions in our trial were similar to those found by Lönnberg et al. (2021) in an *n* = 193 RCT of MBCP vs. Lamaze in Sweden that yielded a 9% rate of PPD in the MBCP group vs. a 29% rate in the Lamaze group at 3 months postpartum. Reducing postpartum mood disturbance is of critical importance for early parenting. Depressed mothers show decreased neural activation in response to their own infant’s cries (aligned with behavioral inhibition of appropriate responses to infant cues) compared to non-depressed mothers (Laurent & Ablow, 2012). What is more, PPD is linked with long-term negative effects on child developmental outcomes (Pearlstein et al., 2009). The MBCP RCT in Sweden (Lönnberg et al., 2021) included parenting and child outcomes. They found a significant effect of MBCP on parent-reported child social-emotional development compared to the Lamaze group. Future directions for CP/CP + include longitudinal follow-up of families.

We found a trend toward lower postpartum anxiety in the CP + group when controlling for baseline prenatal anxiety. A recent noninferiority trial of MBSR suggests that mindfulness training can be as effective as psychotropic medication for the treatment of anxiety disorders (Hoge et al., 2023). Given uncertainty about the effects of medications on fetal and child development, and potential for overestimating risks of medication use (Nordeng et al., 2010), pregnant and postpartum people may wish to avoid pharmacotherapy. During pregnancy, for example, exposure to selective serotonin reuptake inhibitors (SSRIs), serotonin-norepinephrine reuptake inhibitors (SNRIs), and second-generation antipsychotics (SGAs) has been shown to be related to elevated risk for poor neonatal adaptation syndrome (Viguera et al., 2023). Earlier studies showed adverse effects of fluoxetine, citalopram, doxepin, bupropion, and nefazodone in infants who were breastfeeding, yet untreated PPD has comparable adverse effects and other risks such as suicide and infanticide (Pearlstein, et al., 2009). Avoiding medication requires effective alternative treatment options; evidence is accumulating that mindfulness training may be a viable alternative for some mental health conditions.

Other hypothesized secondary outcomes on psychological well-being were only partially supported, with statistically significant differences found between the two groups only for people with better baseline levels of functioning on mindfulness, depression symptoms, or negative emotion. The sample size here was quite small for moderation analyses and should be interpreted with caution. These findings contrast with a systematic review and meta-analysis of perinatal MBPs for pregnant women with and without mental health issues that showed greater improvement for those with worse mental health (Yan et al., 2022). It may be that receipt of care occurred too late in pregnancy to reduce the risk presented by poor prenatal psychological well-being. The average gestational age in weeks of participants at baseline assessment was 19.67 weeks (*SD* = 3.85). There may also have been limitations to our measurement approach. Although we used measures validated in Spanish, interviewer notes from the study visits indicate that our conceptualization of stress and coping was a mismatch for some participants’ beliefs and cultural norms around not naming pregnancy-related anxiety or stress and instead focusing on a positive, faith-based approach to viewing pregnancy as a blessing. This contrasts with provider reports of prenatal anxiety in our pre-pilot survey.

The MBCP additions to CP + are also low-dose compared to the full 9-week course, with short in-session practices, and gentle invitations to practice mindfulness outside of group when possible, using guided audio instructions ranging in duration from approximately 6 min of breath awareness practice to 27 min of mindful movement practice. In contrast, MBCP participants are encouraged to practice mindfulness meditation for 30 min per day, 6 days per week for 9 weeks, similar to the 8-week MBSR and MBCT programs. Other low-dose mindfulness programs have shown benefit on depression (Virgili, 2015; Xia et al., 2022), as we saw here, but it may be that more practice is needed to shift the other indicators. One meta-analysis examining the dose–response relationship between MBPs and psychological outcomes showed MBPs were beneficial, yet dose did not robustly influence outcomes for depression, anxiety, and stress, whereas MBP doses related to program intensity, actual program use, and facilitator contact were key to improvements in mindfulness (Strohmaier, 2020). In our study, although participants were receptive to the mindfulness content during their group sessions, they often reported substantial barriers to practicing mindfulness outside of their CP + groups, e.g., due to the lack of access to a private and quiet space to practice. Unfortunately, we do not have sufficient data on participant group session attendance or home practice to report those rates or conduct dose–response analyses. These gaps in our data represent an important limitation of the study and are areas for future investigation, particularly when adopting a fully pragmatic trial design vs. the intent-to-treat analysis approach taken here.

Many of the participants eligible for the study were experiencing low or very low income and either homelessness (e.g., sleeping in a van in a city park) or other forms of unstable housing (e.g., temporarily renting a room in a crowded apartment with multiple other families). These factors significantly contributed to the stress burden among participants as well as greatly added to the challenge of meeting the study’s target enrollment goal, thus limiting power to test study hypotheses. Contact information for participants (address, telephone number) changed frequently, if it was available at all. Participants often needed to reschedule visits numerous times, and often lost contact when their prepaid cell phone minutes expired. Baseline and 3rd trimester visits were limited to the operating hours of the CRC where they were held, further reducing opportunities for study scheduling. Despite superb research staff outreach efforts, support from clinical staff, and generally high receptivity from potential participants, the sample of *n* = 49 took longer than planned to recruit and enroll and ultimately limited the statistical power to test study hypotheses. We were also unable to collect data on (a) the small number of people who declined to participate in CP at our partner site prior to referral to our study for enrollment screening, or (b) the rate of partner/support person inclusion in CP/CP + groups. However, much was learned about the feasibility of the approach, with room for improvement in future implementations of mindfulness skills training in prenatal group visits. Additionally, the need for more structural supports and public services for pregnant people living through such trying conditions is underscored by this study. It may be that mindfulness training is understandably swamped by other concerns about meeting basic survival needs.

Our partner sites—the midwifery service of the safety net public hospital and the community-based organization—were focused on linking participants with needed services around housing, food security, legal support, and healthcare. Ninety-two percent of our sample received Medicaid vs. a national average of 41% of women giving birth in 2021 (Osterman et al., 2023). Both groups (CP and CP +) received equivalent access and support for utilizing those services. We saw group differences at baseline in family income with the CP + group reporting higher income than the CP group. We controlled for both family income and baseline levels of outcomes in our analyses per clinical trial analysis guidelines, yet substantially lower family income likely has qualitatively different detrimental impacts on multiple dimensions, particularly in the very high cost of living area of the study, that are unaccounted for by simple indicators. Ongoing research is testing the provision of cash transfers (e.g., child tax credit or universal basic income) to families with young children and their impact on child and family well-being long-term. A natural experiment with *n* = 1266 participants demonstrated better outcomes, including mental health, physical health, and financial well-being, in adulthood 20 years later for people whose families had received cash transfers when they were children (Copeland et al., 2022). Until systems can provide everyone with the basic levels of resources needed for sustaining a healthy and safe life for their new babies, it is to be expected that experiencing very low income during the prenatal period would lead to distress that group care and mindfulness are sorely insufficient to ameliorate. That said, we did hear from participants that mindfulness helped them to cope with stressful life events in ways that may have made accessing other services easier.

There were indications of receptivity to the mindfulness additions evidenced in the participants’ rating of the MBCP elements and in their open-ended responses. Retention was nearly equivalent in the two conditions, i.e., CP + = 20/25 (80%) and CP = 19/24 (79.2%), with slightly lower rates of attrition than found in a systematic review of mindfulness-based perinatal interventions (Leng et al., 2023) that calculated average attrition across studies as 22.5% for the experimental groups and 23.2% for the control groups. In our study, satisfaction with care and birth experiences was high across the two conditions. Participants particularly appreciated that their labor and delivery experiences involved a low level of medical intervention (Liu et al., 2017). It was a consideration when planning the study that the mindfulness components may not be well-received by participants, and these indications suggest that it was feasible and did not undermine satisfaction with care offered through CP in a midwifery care context. Thus, the study met at least one criterion of non-inferiority that is desirable when adapting an evidence-based program that could be further tested in an equivalence or non-inferiority trial. The safety net public hospital setting was somewhat unique in that it prioritizes midwifery care and CP as options for pregnant people in an effective partnership with obstetrics, which could serve as a model for prenatal care more broadly (Hutchison et al., 2011).

Although further research on CP + is certainly needed following this small pilot, when considering dissemination of this model, provider training is a central factor. MBCP facilitator training requires a depth of personal mindfulness practice (in alignment with certification criteria for other major MBPs) that was not required in the current study. It is unknown if facilitating lower dose MBPs, such as CP +, with fidelity requires the same level of professional training and personal mindfulness meditation practice as MBCP, MBSR, or MBCT. CP providers are trained by the Centering Healthcare Institute in “facilitative leadership,” an essential element of the CP model that is in alignment with mindful listening. One study of CP demonstrated that process fidelity to using a facilitative leadership style contributed more to CP effects on birth outcomes than content adherence to discussion topics (Novick et al., 2013). Full-dose MBCP professional training may be needed to prepare to deliver CP + most effectively and may lead to better CP fidelity (even in the absence of offering MBCP content for participants) through strengthening the facilitative group process dimension. It may be that facilitative leadership was the active mechanism of change in our trial, leading to some better outcomes in the CP + group due to provider mindfulness training. We were, however, unable to assess this.

Following completion of study data collection, we provided a daylong mindfulness training for CP providers and group co-leaders from our partner sites. In an evaluation of the training, they reported their enthusiasm for adopting the CP + model and for more training to do so. When asked about key takeaways, several respondents noted things like, “I need to prioritize my own mindfulness practice.” They were also far more interested in learning about how to implement the specific MBCP practices than about the research behind the approach. Since that time, CHI has added some mindfulness elements to the CP curriculum and our partner sites are continuing to implement some aspects of CP +. The midwifery service has also increased the number of CNM providers who identify as Latina and has begun CP groups for Black-identified pregnant people offered by Black-identified CNMs, which offers more racial/ethnic concordance between BIPOC patients and their prenatal care providers.

In the current study, the White investigators and CNMs lacked racial/ethnic concordance with the predominantly Latina BIPOC participants who made up most of the sample, which was only 10% White. Study staff and group co-leaders, some of whom identify as Latina, had greater concordance and ability to meet linguistic needs; however, no providers or study staff identified as Black or Indigenous. Some of the Black women in the SF Bay Area study emphasized their strong preference for receiving care from BIPOC providers (Kemet et al., 2022). Two participants identified as LGBTQ +, a population that commonly experiences deficits in competent and respectful prenatal care (Kukura, 2022); our partner site is known for providing high-quality care for LGBTQ + people. For both CP + and MBCP, additional linguistic and cultural adaptation is also likely needed both for intervention content and measurement tools. For example, when the study was being planned, we used “atención plena” as the Spanish-language translation of the term “mindfulness.” More recent work suggests that “atención consciente” or “conscious attention” provides a better translation as it captures the awareness dimension of the construct (Ibinarriaga Soltero, 2021; Ibinarriaga Soltero et al., 2023). Recent recommendations for culturally responsive MBPs for African Americans include using African American facilitators, culturally familiar settings, and incorporating cultural values and terminology (Watson-Singleton et al., 2019). Across healthcare in the U.S., much work is needed to address inequities and as Carter, the EleVATE Women Collaborative, and Mazonni (2021; p. 108) have pointed out, “group prenatal care alone will not dismantle structural racism. Healthcare institutions need to name ‘racism’; change discriminatory policies; disaggregate data by race and ethnicity to identify inequities; and reallocate time, money and personnel in pursuit of this effort.”

On the continuum of pragmatic trials to explanatory researcher-controlled trials, our study was closer to the real-world pragmatic end (Patsopoulos, 2022); however, it could have been greatly strengthened using community-based participatory (CBPR) or participatory action research (PAR) frameworks. We gathered CP provider input through the survey conducted prior to developing, implementing, and testing CP +; however, we did not adequately engage affected communities in voicing their needs and desires regarding integrating prenatal healthcare and mindfulness training beyond the pre-pilot groups conducted in English. Ongoing and pending collaborative work is oriented toward following the lead of Latina and other BIPOC community leaders, community health workers, and doulas advancing maternal-child health equity using principles from liberation psychology.

CP is currently available in 44 states and territories through nearly 500 sites (CHI, 2023). Thus, if CP + proves effective in larger trials, it has a ready dissemination network. There also may be avenues for testing further cultural adaptation and dissemination of the full MBCP program under BIPOC leadership with CBPR and PAR methods. Childbirth education holds great public health potential as it is widely accepted and accessed, with reports of half of pregnant people attending some form of childbirth education annually in the U.S. (Declercq et al., 2013). Childbirth education has shown some positive effects on obstetric-related outcomes in recent research yet does not appear to improve perinatal mental health (Vanderlaan et al., 2023). A strength of the MBCP approach is that it can replace traditional hospital and community-based childbirth education if adequate resources are allocated. An abbreviated version of MBCP is being examined for delivery within the National Health Service in the U.K. with promising preliminary results (Warriner et al., 2018).

Adapting evidence-based interventions must be undertaken with care. For mindfulness programs, Loucks et al. (2022) have provided guidelines for doing so with integrity, with which our approach aligns. Ultimately, if the mindfulness field is to move toward greater inclusivity at minimum, and liberatory practice in the face of systemic injustice as a visionary intention, we must follow the leadership of BIPOC scholars such as Michael Yellowbird on “Decolonizing Mindfulness,” (Yellow Bird, 2016), Rhonda Magee on “The Inner Work of Racial Justice” (Magee, 2019), and Natalie Watson-Singleton, Angela Rose Black, and Mindfulness for the People on mindfulness by and for Black women (Watson-Singleton & Black, 2022), among others. Their work has informed our interpretation of the results of this small study and a reimagining of how our future directions may better serve expectant parents and the babies yet to be born, to whom we offer the wishes from the CP + loving-kindness meditation:

“May you be safe and protected, Que esté a salvo y protegidx,

May you be happy, Que sea feliz,

Maybe you be healthy, Que esté sanx,

May you live with calm and in peace, Que viva con

tranquilidad y en paz.”

## Supplementary Material

Supplementary Material

## Figures and Tables

**Table 1:** Participant demographic characteristics

	CP (*n* = 24)	CP + (*n* = 25)

Age in years [*M* (*SD*)]	26.23 (4.03)	26.50 (4.77)
Gender: female [*n* (%)]	24 (100)	25 (100)
Sexuality [*n* (%)]		
Heterosexual or straight	22 (91.7)	25 (100)
Gay or lesbian	1 (4.2)	0
Bisexual	1 (4.2)	0
Race/ethnicity [*n* (%)]		
American Indian/Alaskan Native/Native American/Indigenous	1	3
Black/African American	3	1
Latin(a), Latin American, or Hispanic	16	16
White/European-American	1	4
Multi-racial	3	1
Education [*n* (%)]		
4 years or less	1 (4.2)	0
High school degree or equivalent	4 (16.7)	10 (40)
Associate’s degree	3 (12.5)	1 (4)
Bachelor’s degree	3 (12.5)	3 (12)
Other	2 (8.3)	1 (4)
5–11 years of education	11 (45.8)	10 (40)
Household income [*n* (%)]		
Less than US$10,000	12 (54.5)	4 (22.2)
US$10,001–$30,000	7 (31.8)	7 (38.9)
US$30,001–$50,000	2 (9.1)	3 (16.7)
US$50,001–$70,000	1 (4.5)	3 (16.7)
US$70,001–$90,000	0	1 (5.6)
Languages [*n* (%)]		
Spanish	16 (66.7)	15 (60)
English	8 (33.3)	10 (40)
Birth country		
Belgium	0	1 (4)
El Salvador	1 (4.2)	1 (4)
Guatemala	3 (12.5)	3 (12)
Honduras	2 (8.3)	1 (4)
Mexico	5 (20.8)	10 (40)
Nicaragua	2 (8.3)	1 (4)
Peru	2 (8.3)	0
United States	9 (37.5)	8 (32)
Years in the U.S. [*M* (*SD*)]	5.41 (2.23)	7.71 (6.41)
Housing [*n* (%)]		
Apartment/house	15 (62.5)	16 (64)
Homeless	1 (4.2)	1 (4)
Other (e.g., renting a room)	8 (33.3)	8 (32)
Receipt of social services (*n*)		
Medicaid/Medicare	24	21
Healthy San Francisco/Healthy	4	4
Families		
Food stamps/EBT	10	4
WIC	19	21

**Table 2:** Sample Outline of CenteringPregnancy with Mindfulness Skills (CP +)

CP + session	MBCP element added

1	Orientation to mindfulness for pregnancy, childbirth, and parenting Mindful Raisin exercise Home practice invitation: Mindful Eating
2	Review mindful eating home practice Mindfulness for physical challenges of pregnancy (“being with” mode) Centering Breathing Space (mindful breath awareness) Mindful Stretching and Being with Baby practice Home practice invitation: Mindfulness of activities of daily living Continue mindfulness of food choices Encourage use of guided mindfulness audio practices (CD/mp3)
3	Opening practice: Centering Breathing Space Being with Baby Practice Review home practices: mindful activities of daily living and use of guided audios Discussion of depression with mindfulness thread (“I am not my thoughts”) Body Scan Practice Home practice invitation: Same as weeks 1 and 2, plus choice of Mindful Movement/Yoga, Body Scan or Centering Breathing Space
4	Opening practice: Centering Breathing Space Contractions of Labor and Contractions of Life: Mindfulness practices for the pain of childbirth and the stress of daily life Ice Practice: Using mindfulness for working with strong physical sensations (labor preparation) Home practice invitation: Centering Breathing Space, Mindful Movement/Yoga, or Body Scan audios
5	Opening practice: Centering Breathing Space Breastfeeding, Stress, and Mindfulness Home practice invitation: Continue using Ice Practice and mindfulness audios
6	Opening practice: Centering Breathing Space Encouragement to continue mindfulness practice in daily life, exploration of personal experiences with mindfulness practice Process emotions about childbirth using mindfulness lens Families of origin: Parenting mindfully conversation
7	Opening practice: Centering Breathing Space Review Being with Baby practice Working with sensations of labor using mindfulness Lovingkindness Meditation (LKM) adapted to focus first on baby, then self, then CP + group) Home practice invitation: Encouragement to practice, including LKM
8	Opening practice: Being with Baby Practice Lovingkindness Meditation (adapted) Infant care with mindfulness/mindful parenting Home practice invitation: Encouragement to practice, including LKM
9	Opening practice: Offer choice of Lovingkindness Meditation or Centering Breathing Space If some participants have already given birth and babies are present, incorporate mindfulness of sounds of babies in the room Mindfulness skills for the postpartum period (being in “baby time,” mindful parenting, mindfulness for self-care Home practice invitation: Mindfulness/being with baby practices for those who have delivered, other practices to prepare for labor for those who have not yet delivered Close with Lovingkindness Meditation or Centering Breathing Space (whichever not selected for opening)
10	Opening practice: Mindful Walking (with baby) Revisit breastfeeding with mindfulness lens (moment-by-moment, nonjudgmental awareness) Processing birth experiences, including conversation of mindfulness practices used during labor Home practice invitation: Mindfulness with Baby for those who have delivered, other practices to prepare for labor for those who have not Close with Lovingkindness Meditation or Centering Breathing Space

Other CP discussion topics for each session are not listed

**Table 3:** Means and standard deviations of study variables across three time points

	CP (*n* = 24)		CP + (*n* = 25)	
Variable	Mean	*SD*	Mean	*SD*

Postpartum depression (EPDS) T3	8.68	5.39	4.90	4.06
Cases with EPDS	8 cases		2 cases	
*score* ≥ 10 at T3				
Trait Anxiety T3	40.79	9.06	35.80	7.81
Mindfulness				
T1	3.28	0.39	3.31	0.35
T2	3.27	0.45	3.32	0.34
T3	3.26	0.34	3.39	0.43
Depression symptoms (CES-D)				
T1	19.67	11.69	16.60	8.87
Cases with CES-D	13 cases		11 cases	
*score* ≥ 16 at T1				
T2	18.95	11.23	13.89	8.72
Cases with CES-D	12 cases		4 cases	
scores ≥ 16 at T2				
T3	16.63	9.79	11.65	8.71
Cases with CES-D	10 cases		4 cases	
scores ≥ 16 at T3				
Perceived stress				
T1	17.67	8.59	16.88	5.29
T2	16.05	7.01	14.33	4.80
T3	16.84	6.66	13.90	5.05
Positive emotions				
T1	33.42	6.57	32.64	7.40
T2	30.58	7.27	30.44	7.38
T3	34.95	6.83	34.30	8.39
Negative emotions				
T1	20.50	10.18	16.96	6.82
T2	20.89	10.76	16.44	9.01
T3	16.47	8.64	12.15	7.75
Pregnancy anxiety				
T1	25.38	8.13	25.72	6.65
T2	23.90	8.25	23.61	6.27
Birth satisfaction T3	9.26	1.94	8.70	0.97

Time 1 = T1; Time 2 = T2, and Time 3 = T3. *EPDS* Edinburgh Postnatal Depression Scale, *CES-D* Center for Epidemiologic Studies Depression Scale

**Table 4:** Perceived benefits of adapted MBCP-related elements of CP +

Quantitative item	3rd trimester		Post-birth	
*“Practicing ____ has helped me to feel less stress.” (0 = Strongly Disagree, 3 = Strongly Agree)*	*M*	*SD*	*M*	*SD*

Mindfulness practice in group	2.47	0.72	2.37	0.60
Centering breathing space	2.43	0.51	2.11	0.47
Mindful movement yoga	2.33	0.49	2.21	0.70
Body scan	2.27	0.47	2.07	0.59
Mindful pain coping (preparation for labor)	2.12	0.78	2.37	0.76
Qualitative item	Representative responses			
What was the most helpful thing you learned for having a *healthy pregnancy*?	“How to reduce my stress with the breathing and how to not let little things get			
	“Being present in my mind and my body.”			
	“Being absolutely centered and present.			
	Makes it easier to not panic when you can assess what is happening in that moment.”			
What was the most useful thing you learned for *taking care of a new baby*?	“How to stay calm when I can’t stop the baby from crying.”			
	“The baby’s signals that you can tell from their face.”			
	“That there’s no perfect way to do it.”			

## Data Availability

The study was conducted under a Certificate of Confidentiality from the National Institutes of Health. Deidentified data may be available from the corresponding author upon reasonable request.
