# Supplementary material for: Enhancing Prenatal Group Medical Visits with Mindfulness Skills: A Pragmatic Trial with Latina and BIPOC Pregnant Women Experiencing Multiple Forms of Structural Inequity
Source: Mindfulness (N Y). Author manuscript; Available in PMC 2025 Mar 27. (PMC11949468; doi:10.1007/s12671-023-02227-z)
Supplement: Supplementary Material [file NIHMS2021677-supplement-Supplementary_Material.docx]

**Online Supplemental Materials, Figure S1.** Plots of participants’ baseline score as a moderator conditioning the unstandardized effect of group differences in dispositional mindfulness, depression symptoms, perceived stress, and negative emotions.


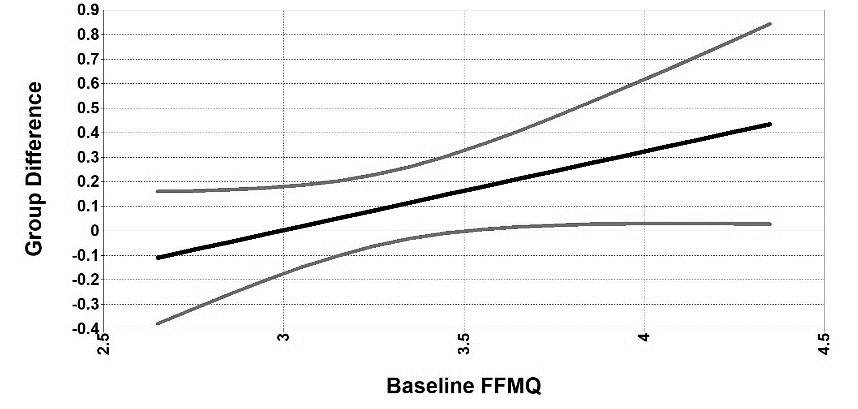

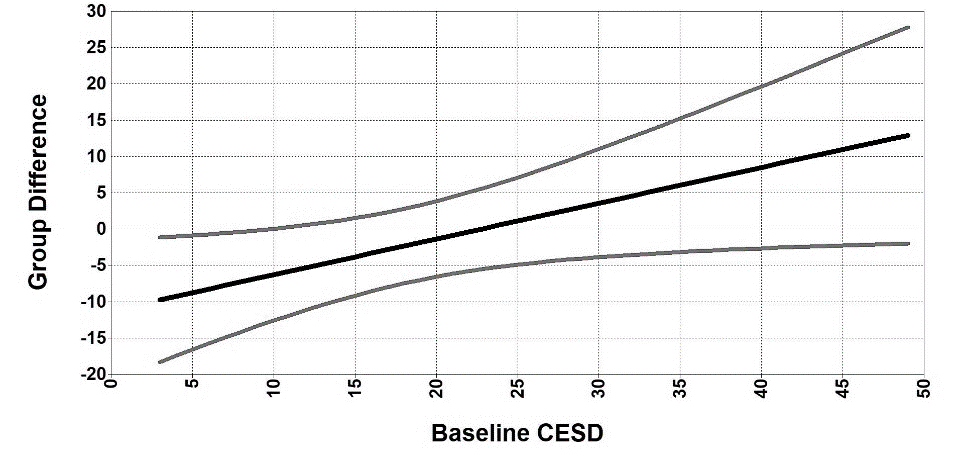

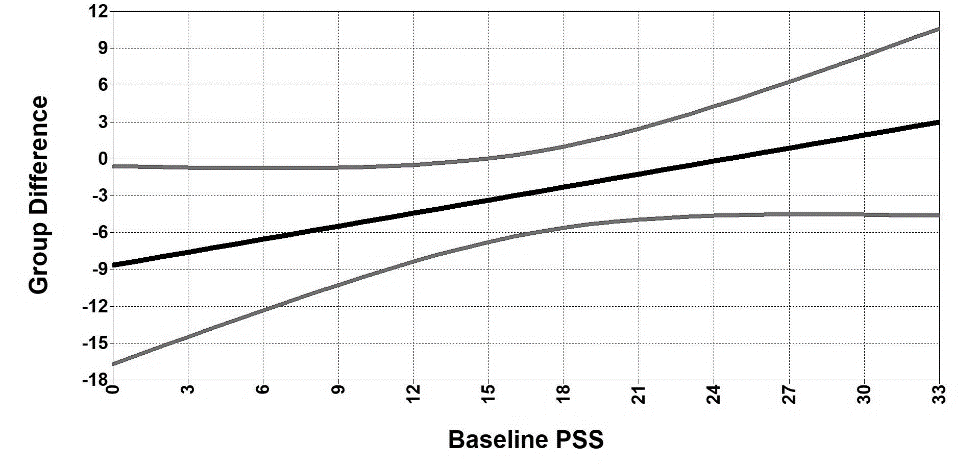

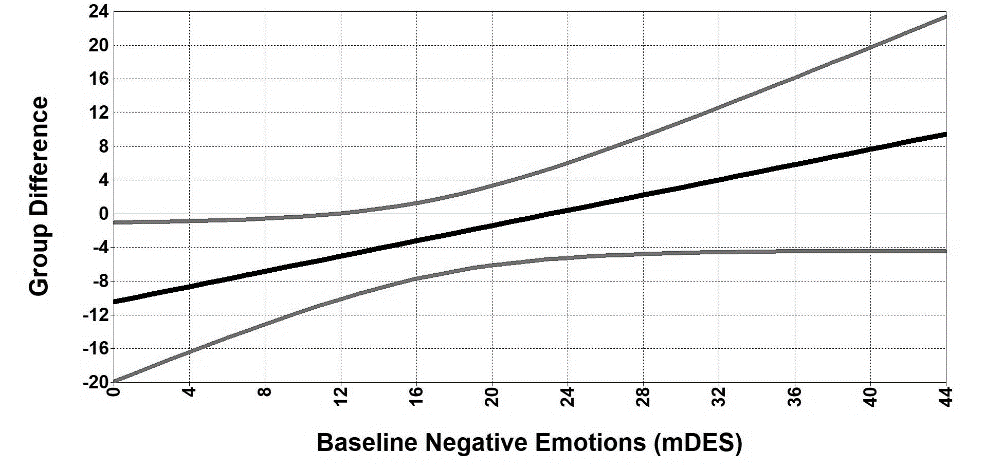


Note: In each region of significance figure, the *x*-axis is participants’ baseline score. The *y*-axis is the unstandardized coefficient of group difference. Curved lines are 95% Confidence Intervals. The horizontal line at *y* = 0 denotes there was no significant group difference. Grey-shaded area is region of significance, suggesting that the CenteringPregnancy with Mindfulness Skills (CP+) program showed significant improvements on the outcome compared to the CenteringPregnancy program for participants with the selected range of baseline scores.

**Online Supplemental Materials, Table S1.** Results from regression models testing group differences moderated by baseline.

| Variable name | Estimated effect of  group assignment | | Estimated effect of baseline level | | Estimated effect of  group x baseline | |
| --- | --- | --- | --- | --- | --- | --- |
|  | B | β | B | β | B | β |
| Dispositional Mindfulness | -0.958 | -1.260 | 0.704******* | 0.674 | 0.320**+** | 1.409 |
| Depression | -11.223***** | -0.613 | 0.221 | 0.247 | 0.493***** | 0.558 |
| Perceived Stress | -8.663***** | -0.746 | 0.329****** | 0.395 | 0.353^a^ | 0.560 |
| Positive Emotions | 0.412 | 0.027 | 0.672****** | 0.611 | -0.040 | -0.091 |
| Negative Emotions | -10.449***** | -0.644 | 0.381****** | 0.406 | 0.453**+** | 0.543 |
| Mind-Body Coping (from WOC) | -0.020 | -0.013 | 0.376 | 0.273 | 0.080 | 0.051 |
| Pregnancy Anxiety (3^rd^ trimester) | 0.751 | 0.054 | 0.786 | 0.818 | -0.091 | -0.179 |

Note: All variables were assessed post-birth other than pregnancy anxiety which was assessed in the 3^rd^ trimester.

*** *p* < 0.001. ** *p* < 0.01. * *p <* 0.05. + *p* < 0.10. Family income was controlled for in all models.

^a^*p* = 0.11.
